# A preliminary study of phosphodiesterases and adenylyl cyclase signaling pathway on red blood cell deformability of sickle cell patients

**DOI:** 10.3389/fphys.2023.1215835

**Published:** 2023-09-15

**Authors:** Evrim Goksel, Elif Ugurel, Elie Nader, Camille Boisson, Ingrid Muniansi, Philippe Joly, Celine Renoux, Alexandra Gauthier, Philippe Connes, Ozlem Yalcin

**Affiliations:** ^1^ Research Center for Translational Medicine (KUTTAM), Koc University, Istanbul, Türkiye; ^2^ Department of Physiology, School of Medicine, Koc University, Istanbul, Türkiye; ^3^ Graduate School of Health Sciences, Koc University, Istanbul, Türkiye; ^4^ Laboratoire Interuniversitaire de Biologie de la Motricité (LIBM) EA7424, Team “Vascular Biology and Red Blood Cell”, Université Claude Bernard Lyon 1, Lyon, France; ^5^ Laboratoire d’Excellence du Globule Rouge (Labex GR-Ex), PRES Sorbonne, Paris, France; ^6^ Institut Hématologique et Oncologique Pédiatrique (IHOPe), Lyon, France

**Keywords:** sickle cell disease, deformability, shear stress, adenylyl cyclase, phosphodiesterases, protein kinase A

## Abstract

Sickle cell disease (SCD) is an inherited hemoglobinopathy characterized by chronic anemia, intravascular hemolysis, and the occurrence of vaso-occlusive crises due to the mechanical obstruction of the microcirculation by poorly deformable red blood cells (RBCs). RBC deformability is a key factor in the pathogenesis of SCD, and is affected by various factors. In this study, we investigated the effects of adenylyl cyclase (AC) signaling pathway modulation and different phosphodiesterase (PDE) modulatory molecules on the deformability and mechanical stress responses of RBC from SCD patients (HbSS genotype) by applying 5 Pa shear stress with an ektacytometer (LORRCA). We evaluated RBC deformability before and after the application of shear stress. AC stimulation with Forskolin had distinct effects on RBC deformability depending on the application of 5 Pa shear stress. RBC deformability was increased by Forskolin before shear stress application but decreased after 5 Pa shear stress. AC inhibition with SQ22536 and protein kinase A (PKA) inhibition with H89 increased RBC deformability before and after the shear stress application. Non-selective PDE inhibition with Pentoxifylline increased RBC deformability. However, modulation of the different PDE types had distinct effects on RBC deformability, with PDE1 inhibition by Vinpocetine increasing deformability while PDE4 inhibition by Rolipram decreased RBC deformability after the shear stress application. The effects of the drugs varied greatly between patients suggesting some could benefit from one drug while others not. Developing drugs targeting the AC signaling pathway could have clinical applications for SCD, but more researches with larger patient cohorts are needed to identify the differences in the responses of sickle RBCs.

## 1 Introduction

Sickle cell disease (SCD) is an inherited hemoglobinopathy characterized by a point mutation in the β-globin gene, resulting in the production of an abnormal hemoglobin (HbS) (Piel et al., 2017). The polymerization of HbS, which occurs in deoxygenated conditions, causes a mechanical distortion of red blood cells (RBC) resulting in a change of cell morphology into a sickle shape (Rees et al., 2010; Connes et al., 2018). Repeated cycles of sickling and unsickling may damage the RBC membrane, deteriorate deformability, increase cell adhesiveness and ultimately lead to vaso-occlusions, intravascular hemolysis, and chronic anemia (Lamarre et al., 2012; Alapan et al., 2014; Connes et al., 2014; Jang et al., 2021). Stiff and poorly deformable sickle RBCs have detrimental effects on the microcirculation by being trapped in the postcapillary venules and impairing blood flow, which may lead to painful vaso-occlusive crises and organ damages (Eaton and Hofrichter, 1987; Chiang and Frenette, 2005; Conran et al., 2009).

RBC deformability depends on intracellular viscosity, surface/volume ratio, and the cytoskeletal interactions with the integral membrane components, as well as ATP levels and redox state (Huisjes et al., 2018; Kuck et al., 2020). ATP is an important energy source for the proper function of ion channels and essential for the intracellular ion balance to maintain cell hydration (Chu et al., 2012; Leal Denis et al., 2016; Gallagher, 2017). Dehydration and reduced ATP levels have been reported in RBCs from SCD patients (Gulley et al., 1982; Banerjee and Kuypers, 2004, Sabina et al., 2009). The impaired production of ATP in sickle RBCs could adversely affect AC signaling pathway. The activation of signaling molecules and enzymes involved in adenylyl cyclase (AC) pathway is dependent on the conversion of ATP to cAMP. Several studies demonstrated a role of AC signaling pathway in the regulation of the deformability of healthy RBCs (Sprague et al., 2001; Muravyov et al., 2009; Muravyov and Tikhomirova, 2013; Semenov et al., 2019). Indeed, ATP levels could affect AC pathway, which may participate to the decrease in RBC deformability in this disease. We have previously shown the potential role of AC pathway for the modulation of RBC deformability in SCD patients and demonstrated that the inhibition of cAMP hydrolysis by phosphodiesterases increased the deformability of RBCs from SCD patients (Ugurel et al., 2019).

Once the AC enzyme is activated by the G protein coupled receptor (GPCR), it catalyzes the conversion of ATP to cAMP that activates cAMP-dependent enzyme (Protein kinase A, PKA). The signal transduction within the cell is carried out depending upon enhanced cAMP levels, and the signal is terminated when cAMP is converted to AMP by phosphodiesterases (PDE). Hence, PDEs provide negative feedback for the signaling pathways. It has also been shown in SCD mice model that activation of PKA through stimulation of A_2B_R receptor with adenosine promotes cAMP production, leading to RBC sickling (Zhang et al., 2011). Significantly higher RBC cAMP levels have been reported in SCD patients, and correlation with the frequency of painful vaso-occlusive crises has been reported (Hines et al., 2003; Jit et al., 2019). A reduction in cAMP level was also observed in sickle RBCs during hydroxyurea treatment (Bartolucci et al., 2010), a treatment that decrease the risk of vaso-occlusive crises and acute chest syndrome in SCD. Six different PDE types (PDE1, PDE2A, PDE3B, PDE4, PDE5, and PDE9A) have been identified in RBCs to date and are important for the regulation of cAMP or cGMP (Almeida et al., 2008; Adderley et al., 2010; Adderley et al., 2011), however, their distinct roles in the regulation of RBC deformability in SCD are unknown.

In the present study, we hypothesized that RBC deformability from SCD patients is modulated by selective PDE types and AC signaling pathway. We investigated the modulatory effects of AC, PKA, and different PDE types on RBC deformability by incubating SCD RBCs with selective stimulators and inhibitors. Moreover, RBCs undergo various levels of shear stress in the blood circulation that is fundamental for their ability to deform. Shear stress at the physiological level regulates ATP release from RBCs and calcium (Ca^+2^) influx within RBCs, the latter of which could directly stimulate AC through Ca^+2^-calmodulin (Halls and Cooper, 2011; Cinar et al., 2015; Danielczok et al., 2017). Indeed, we also exposed RBCs from SCD patients to prolonged shear stress that is physiologically relevant and evaluated mechanical stress responses of RBCs by the changes in deformability, before and after the application of shear stress and with or without drugs known to modulate AC, PKA and PDE signaling pathways.

## 2 Materials and methods

### 2.1 Patients and controls

Homozygous SCD patients with HbSS genotype (*n* = 7) were included in the study: age = 21.4 ± 16.5 years, HbF = 13.1 ± 6.6%, HbS = 83.3 ± 6.0%, Hct = 24.5 ± 3.2%, RBC number = 2.85 ± 0.39 10^12^/L, MCHC = 347 ± 12.4 g/L, MCV = 87.4 ± 15.9 fl, all under hydroxyurea therapy. The patients were diagnosed and followed at the Sickle Cell Center of the Academic Hospital of Lyon. All patients were at clinical steady state for at least 2 months prior to their inclusion in the study (i.e., no acute episodes of infection, vaso-occlusive crises, acute chest syndrome, stroke, priapism and no blood transfusions in the preceding 3 months). Every donor gave written informed consent before sampling. The study was conducted in accordance with the guidelines set by the Declaration of Helsinki and was approved by the Regional Ethics Committees (CPP Lyon-Est, Hospices Civils de Lyon, L14-127).

### 2.2 Preparation of blood samples

Peripheral blood was withdrawn from antecubital vein of each donor and taken into EDTA vacuum tubes (15 IU/ml). Hematocrit was set to 40% for the experiments with autologous plasma. Blood samples were treated with the stimulators or inhibitors of the enzymes involved in the PKA pathway. Forskolin (10 μM) and SQ22536 (100 μM) were used for the stimulation and the inhibition of AC, respectively. H-89 (10 μM) and Pentoxifylline (10 μM) were used for the inhibition of PKA and PDEs, respectively. For the selective inhibition of PDEs, Vinpocetine (30 μM), Milrinone (20 μM), and Rolipram (10 μM) were used to block the activities of PDE1, PDE3, and PDE4, respectively. All chemical agents were purchased from Sigma-Aldrich Co (MO, United States) and incubated with blood samples at 37 C for 15 min, except Vinpocetine which was incubated for 30 min. A vehicle (DMSO or PBS) was prepared in the same volume (v/v) and studied under the same conditions as a control. After the incubation with the agents, whole blood samples were measured directly. All experiments were performed within 4 h after blood sampling.

### 2.3 Measurements of RBC deformability

RBC deformability was measured by ektacytometry, using the laser-assisted optical rotational cell analyzer (LORRCA MaxSis, Mechatronics, Netherlands) at 37°C. The laser integrated device consists of a rotating and a static cylinder that generate shear stresses. Briefly, 2.5 mL iso-osmolar polyvinylpyrrolidone (PVP) solution (360 kDa, 29.9 ± 0.5 mPa s, Mechatronics, Netherlands) was mixed with 12.5 uL of the blood sample and placed into the measuring chamber between the two cylinders. RBC deformability was measured by applying 9 different shear stresses (0.30, 0.57, 1.08, 2.04, 3.87, 7.34, 13.92, 26.38, and 50 Pa). A diffraction pattern of RBCs was generated by the laser beam traversing the blood sample. An Elongation Index (EI) was calculated from the diffraction pattern collected by the camera of the LORRCA, which reflected RBC deformability, such as: (A–B)/(A + B), with A and B corresponding to the vertical and horizontal axis of a theoretical ellipse fitting the diffraction pattern. The Lineweaver-Burke method was used to calculate the maximum elongation index at infinite shear stress (EImax) and the shear stress required to reach half of this maximum elongation index (SS1/2) (Baskurt et al., 2009). In order to normalize SS1/2, the ratio SS1/2/EImax was calculated (Baskurt and Meiselman, 2013).

### 2.4 Application of prolonged shear stress to blood samples

The effects of prolonged shear stress on the deformability of RBCs treated or not with the different molecules used in this study, were also investigated. Mixed PVP-RBC suspensions were exposed to continuous shear stress of 5 Pa for 300 s using ektacytometry (LORRCA MaxSis, Mechatronics, Hoorn, Netherlands).The shear stress level at 5 Pa corresponds to a physiological shear stress level at arterial walls (Papaioannou and Stefanadis, 2005). RBC deformability was measured before and after the application of shear stress. Data were recorded as curves of EI-shear stresses.

The following experimental procedure was conducted on blood samples with or without chemical agents: (1) RBC suspensions were used to evaluate RBC deformability before continuous shear stress exposure between 0.3 and 50 Pa, (2) after the measurement has been completed, the sample is aspirated from the gap and the cup is cleaned before the replacement of the next sample, (3) the measuring chamber was filled with new suspension, and 300 s of continuous 5 Pa shear stress were applied and, (4) RBC deformability was measured again immediately following the end of the 5 Pa shear stress exposure.

### 2.5 Statistical analysis

Statistical analysis and data presentation using commercial software were performed (Prism, GraphPad Sofware Inc., United States). The results are shown as a mean ± SD. Shapiro-Wilk normality test was applied for all data sets whether they are normally distributed. A non-parametric Wilcoxon test was performed for the matched data sets which were not normally distributed. Deformability measurements were evaluated before and after shear stress application using a two-way ANOVA with repeated measures followed by Bonferroni multiple comparisons test. A *p*-value less than 0.05 was considered statistically significant.

## 3 Results

### 3.1 RBC deformability from SCD patients is improved by the inhibition of adenylyl cyclase and protein kinase A

**FIGURE 1:**
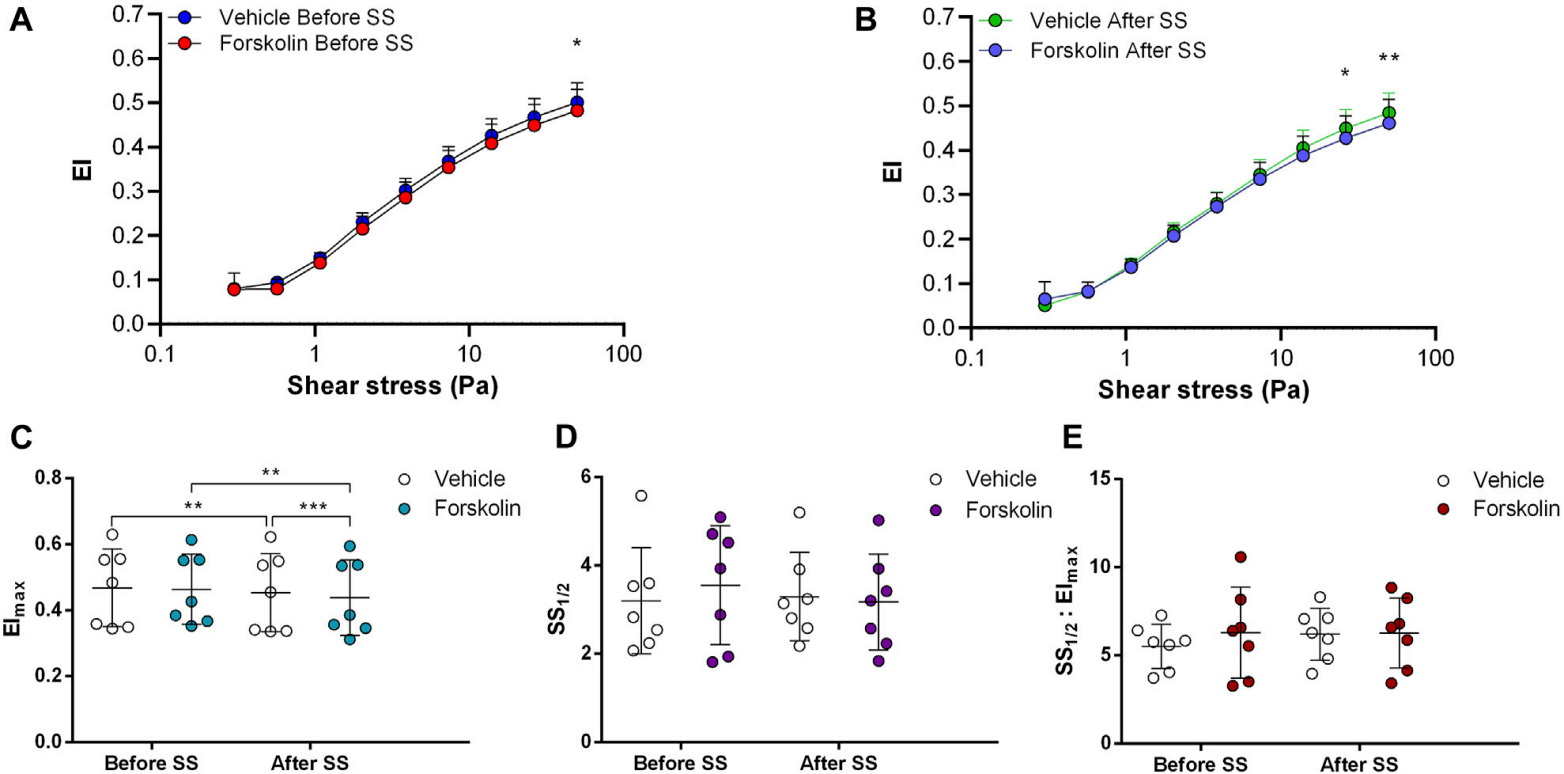
Effects of Forskolin on samples before and after shear stress. Changes in elongation index (EI) before continuous 5 Pa shear stress **(A)** and after continuous 5 Pa shear stress **(B)**, maximum elongation index (El_max_) **(C)**, and the shear stress required to reach half of maximum elongation index (SS_1/2_) **(D)** are shown. El_max_:SS_1/2_ is shown in **(E)**. *n* = 7, ANOVA, *p < 0.05, **p < 0.01, ***p < 0.001.

**FIGURE 2:**
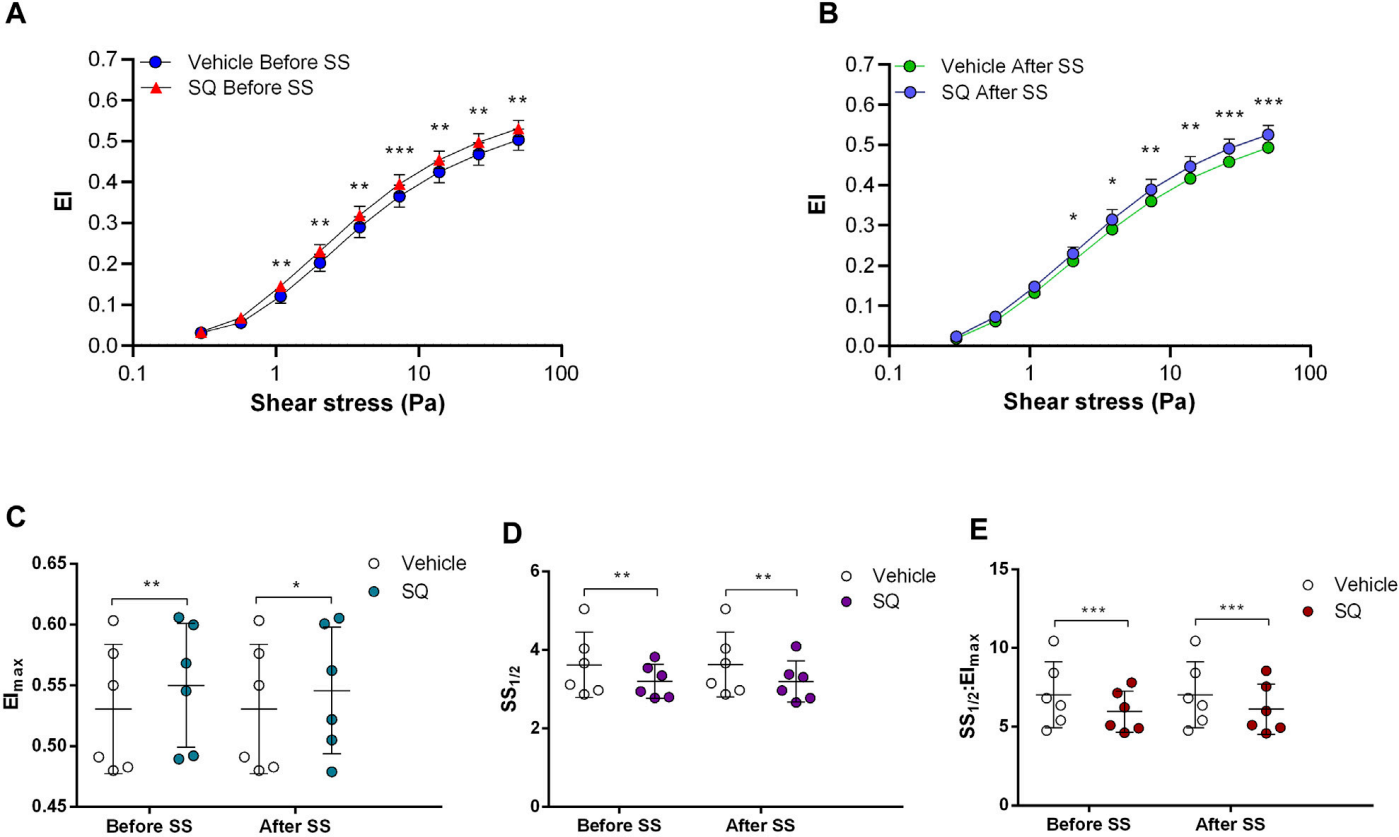
Effects of SQ22536 on samples before and after shear stress. Changes in elongation index (EI) before continuous 5 Pa shear stress **(A)** and after continuous 5 Pa shear stress **(B)**, maximum elongation index (El_max_) **(C)**, and the shear stress required to reach half of maximum elongation index (SS_1/2_) **(D)** are shown. El_max_:SS_1/2_ is shown in **(E)**. *n* = 6, ANOVA, *p < 0.05, **p < 0.01, ***p < 0.001.

**FIGURE 3:**
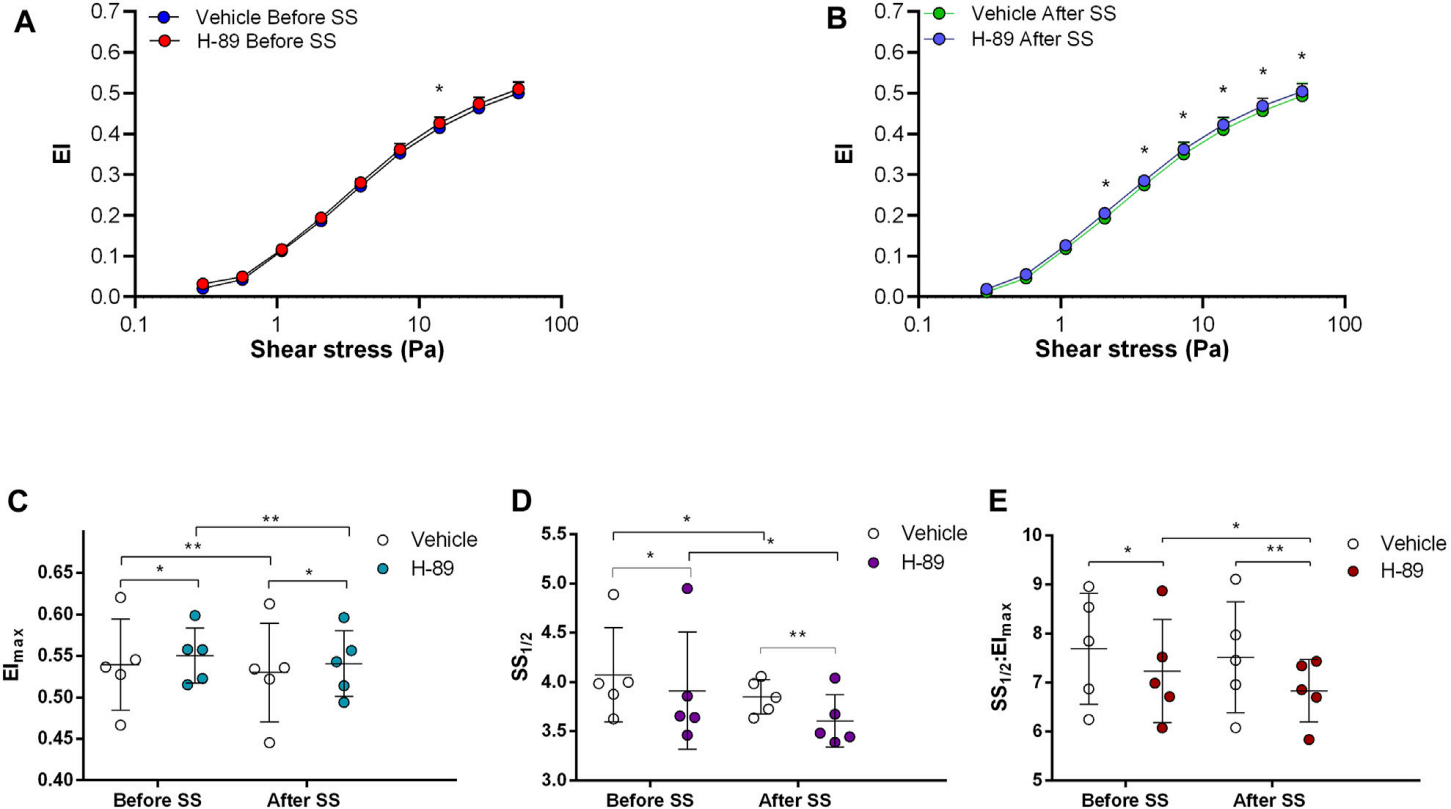
Effects of H-89 on samples before and after shear stress. Changes in elongation index (EI) before continuous 5 Pa shear stress **(A)** and after continuous 5 Pa shear stress **(B)**, maximum elongation index (El_max_) **(C)**, and the shear stress required to reach half of maximum elongation index (SS_1/2_) **(D)** are shown. El_max_:SS_1/2_ is shown in **(E)**. *n* = 5, ANOVA, *p < 0.05, **p < 0.01, ***p < 0.001.

Adenylyl cyclase (AC) stimulation by Forskolin exerted distinct effects on RBC deformability depending on the application of the constant shear stress of 5 Pa. Deformability was improved before the continuous 5 Pa application by 3.7% but deteriorated after by 4.8%, particularly at high shear stress levels (26.38 Pa and 50 Pa) (Figures 1A, B). Interestingly, EImax decreased with Forskolin treatment before and after the 5 Pa application although SS1/2 and SS1/2:EImax values did not change (Figures 1C–E). On the other hand, AC inhibition by SQ22536 resulted in an increase of RBC deformability by 8%–10% between 1.08 Pa and 50 Pa both before and after the constant 5 Pa application (Figures 2A, B). SQ22536 increased EImax values and decreased SS1/2 and SS1/2:EImax values, which indicates a significant increase in RBC deformability (Figures 2C–E). The inhibition of Protein kinase A (PKA) by H89 slightly increased RBC deformability by 2.7% before the 5 Pa application but only at 13.92 Pa level (Figure 3A). After the 5 Pa application, H89 increased RBC deformability between 2.04 Pa and 50 Pa levels by 4% (Figure 3B). EImax values increased and SS1/2 and SS1/2:EImax values decreased with H89 both before and after the application of the continuous 5 Pa shear stress (Figures 3C–E). These results support a modulatory effect of AC/PKA signaling pathway on RBC deformability in SCD.

### 3.2 Different PDE types exert distinct effects on sickle cell deformability

**FIGURE 4:**
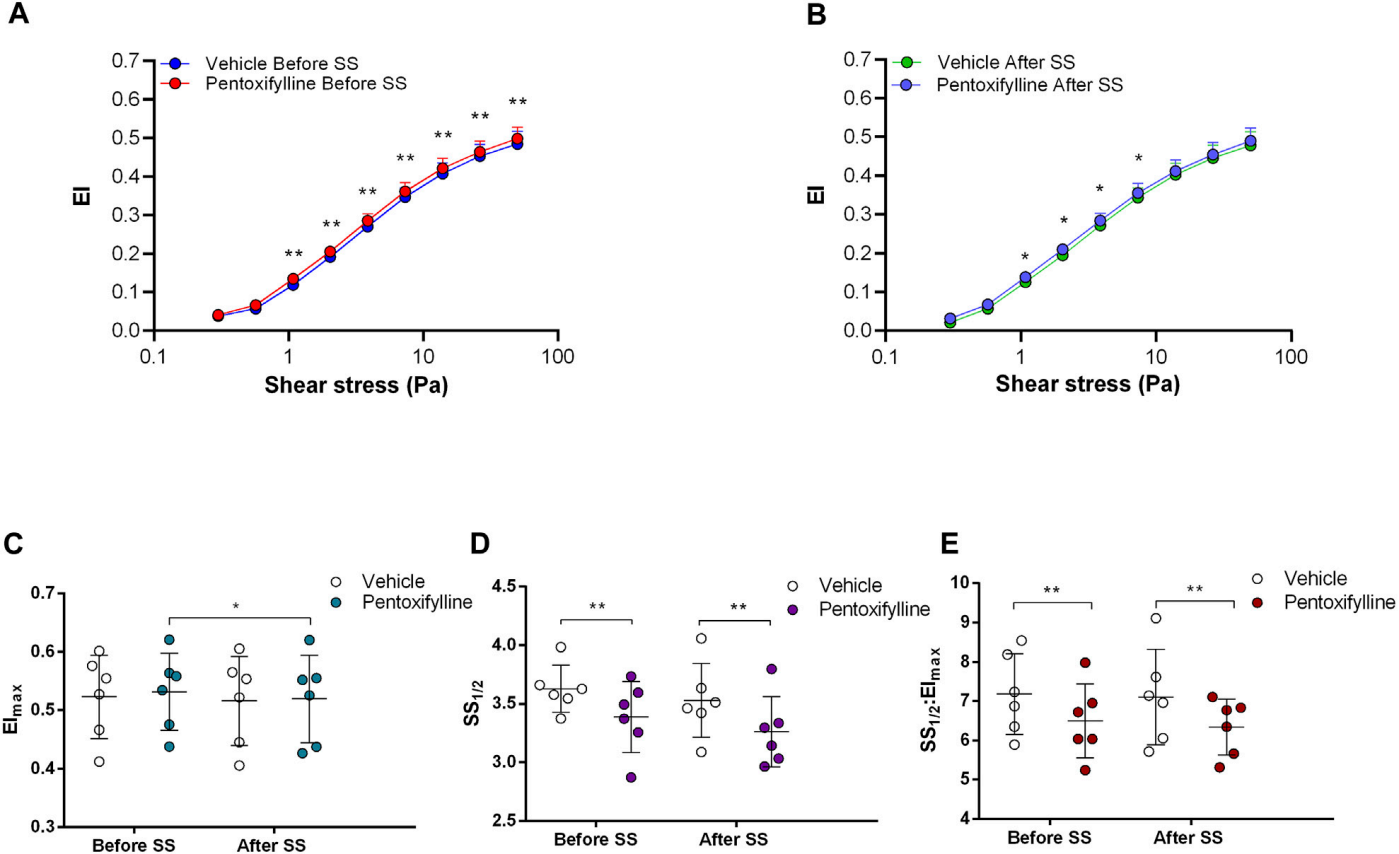
Effects of Pentoxifylline on samples before and after shear stress. Changes in elongation index (EI) before continuous 5 Pa shear stress **(A)** and after continuous 5 Pa shear stress **(B)**, maximum elongation index (El_max_) **(C)**, and the shear stress required to reach half of maximum elongation index (SS_1/2_) **(D)** are shown. El_max_:SS_1/2_ is shown in **(E)**. *n* = 6, ANOVA, *p < 0.05, **p < 0.01.

**FIGURE 5:**
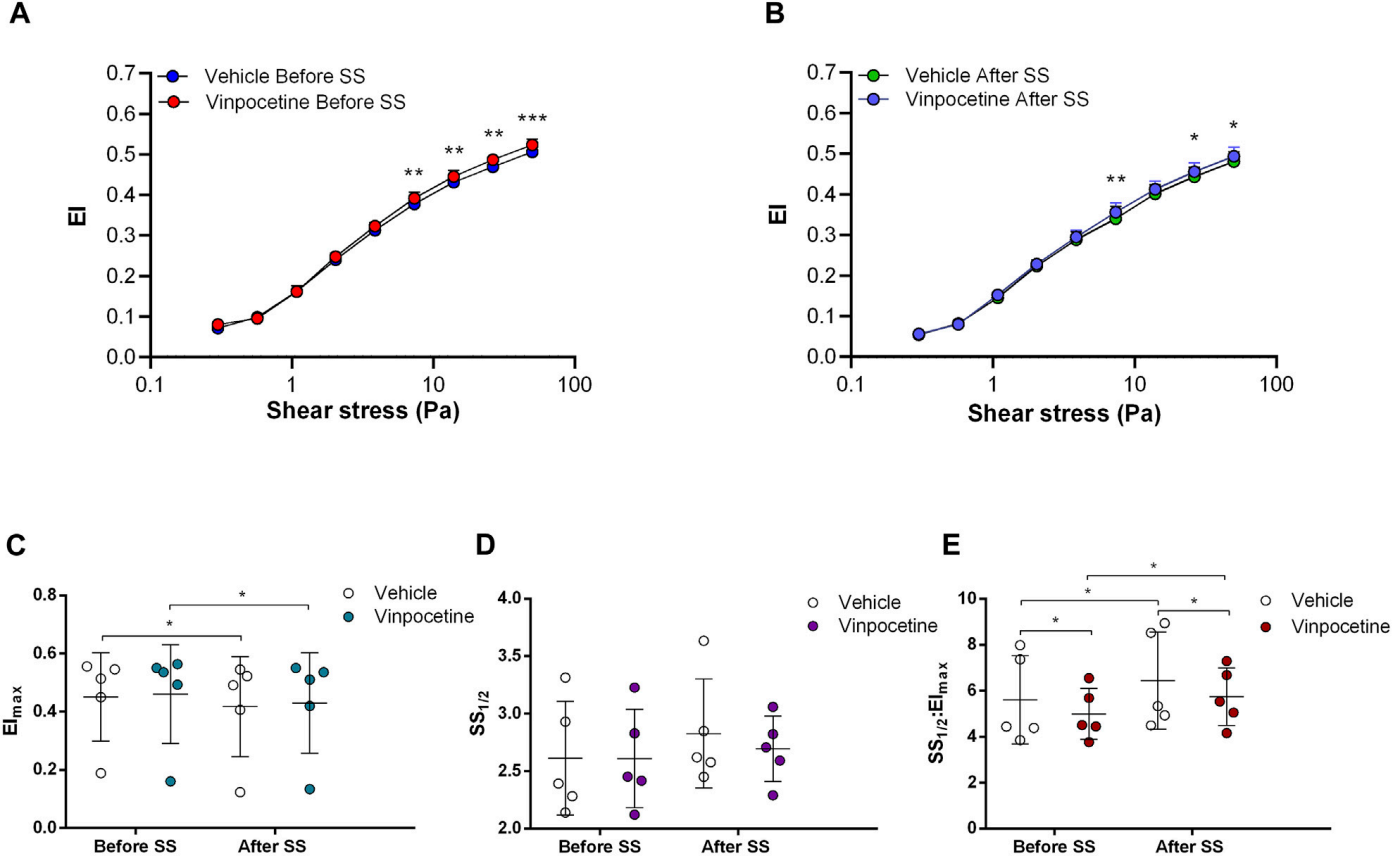
Effects of Vinpocetine on samples before and after shear stress. Changes in elongation index (EI) before continuous 5 Pa shear stress **(A)** and after continuous 5 Pa shear stress **(B)**, maximum elongation index (El_max_) **(C)**, and the shear stress required to reach half of maximum elongation index (SS_1/2_) **(D)** are shown. El_max_:SS_1/2_ is shown in **(E)**. *n* = 6, ANOVA, *p < 0.05, **p < 0.01.

**FIGURE 6:**
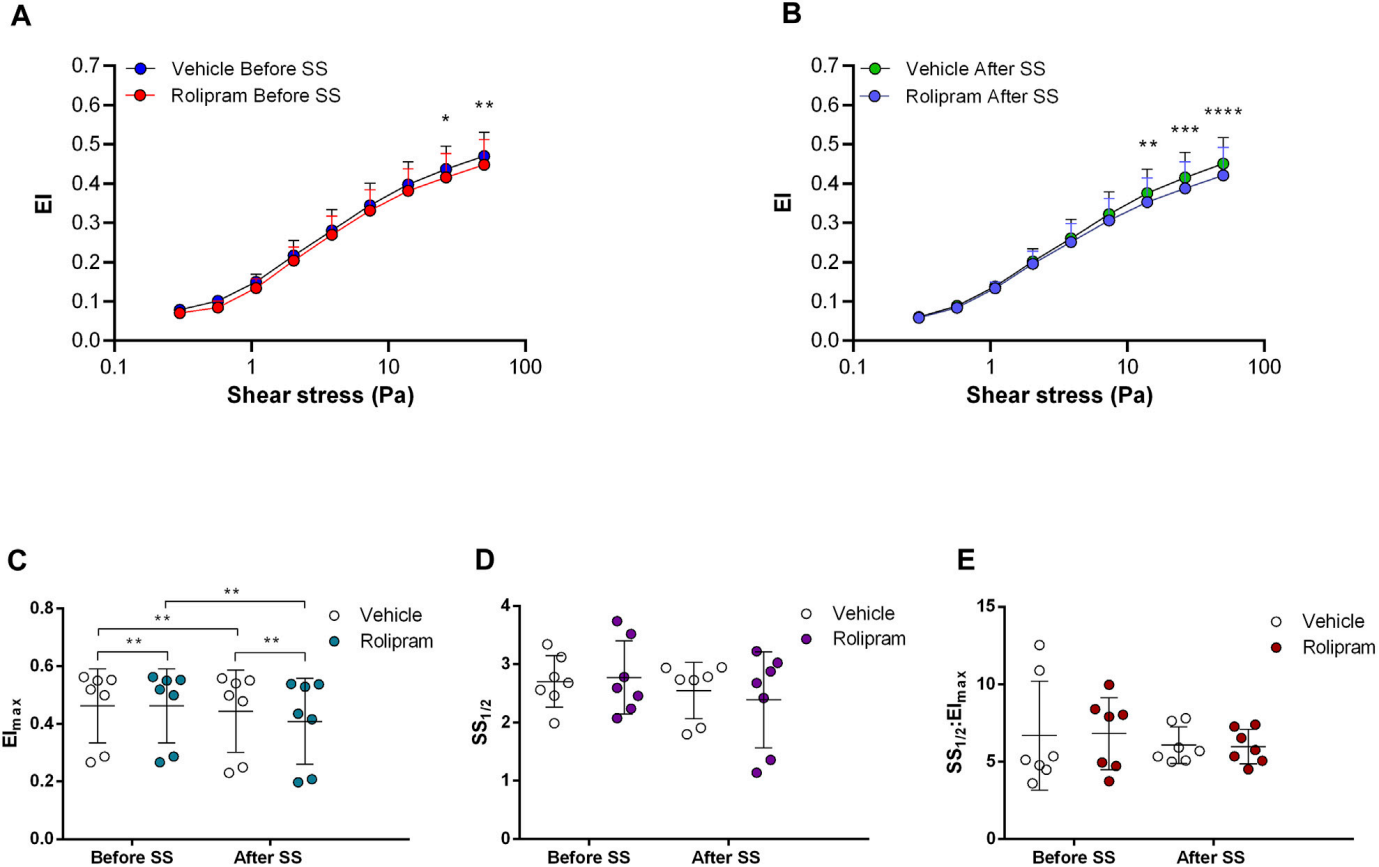
Effects of Rolipram on samples before and after shear stress. Changes in elongation index (EI) before continuous 5 Pa shear stress **(A)** and after continuous 5 Pa shear stress **(B)**, maximum elongation index (El_max_) **(C)**, and the shear stress required to reach half of maximum elongation index (SS_1/2_) **(D)** are shown. El_max_:SS_1/2_ is shown in **(E)**. *n* = 7, ANOVA and Wilcoxon for EI_max_ data, *p < 0.05, **p < 0.01, ***p < 0.001.

**FIGURE 7:**
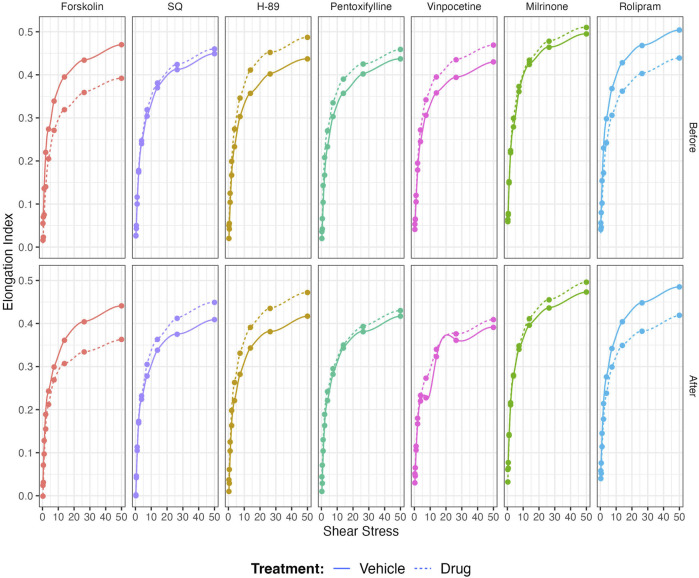
Representative figure of the effects of Forskolin, SQ, H-89, Pentoxifylline, Vinpocetine, Milrinone, and Rolipram on a blood sample collected from a patient with sickle cell disease.

Non-selective inhibition of PDEs by Pentoxifylline resulted in an increase of RBC deformability between 1.08 Pa and 50 Pa by 5.5% before the 5 Pa application and between 1.08 Pa and 7.34 Pa after by 4.7% (Figures 4A, B). EImax did not change but SS1/2 and SS1/2:EImax values significantly decreased with Pentoxifylline both before and after the 5 Pa application (Figures 4C–E). We then investigated the effects of the different types of PDEs on RBC deformability from SCD patients. The inhibition of PDE1 by Vinpocetine significantly increased EI values at high shear stresses (≥7.34 Pa) by 3% both before and after the application of 5 Pa. The inhibition of PDE4 by Rolipram also changed EI values at high shear stresses (≥13.92 Pa). However, Rolipram increased deformability before the 5 Pa application by 4.5% and decreased it after by 6.5% (Figures 5A, B; Figures 6A, B). Vinpocetine decreased SS1/2:EImax ratio but did not change EImax and SS1/2 values (Figures 5C–E). On the contrary, Rolipram did not change SS1/2 and SS1/2:EImax but decreased EImax values both before and after the application of 5 Pa (Figures 6C–E). The inhibition of PDE3 by Milrinone did not significantly affect RBC deformability (data not shown). Figure 7 shows the changes in RBC deformability obtained with the different drugs, before and after the 5 Pa shear stress application, for one SCD patient.

## 4 Discussion

The salient findings in the present study demonstrate the beneficial effects of the inhibitors of adenylyl cyclase (AC)/Protein kinase A (PKA) signaling pathway on the deformability of RBCs from SCD patients. The inhibition of AC and PKA increased RBC deformability before the application of shear stress, while the stimulation of AC decreased it after the 5 Pa shear stress application. To our knowledge, this is the first study investigating the effects of different PDE families on mechanical stress responses of sickle RBCs. Non-selective phosphodiesterase (PDE) inhibition increased RBC deformability. However, blocking particular PDE families produced divergent results showing that the function of the different PDEs is variable in RBCs from SCD patients.

**FIGURE 8:**
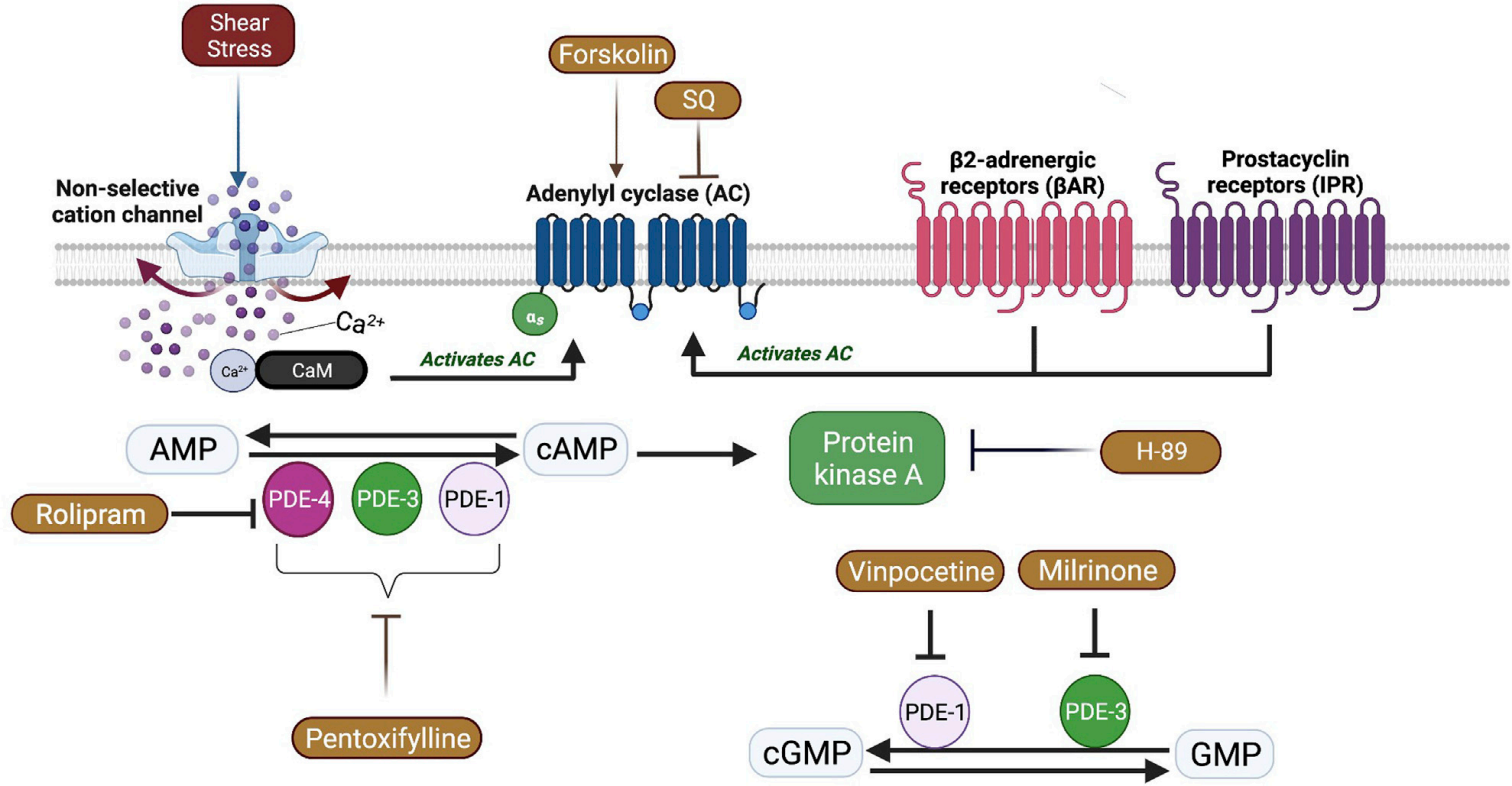
A schematic view of Adenylyl cyclase (AC) signaling pathway and the effects of shear stress in red blood cells. Shear stress could activate some non-selective cation channels which causes calcium (Ca^+2^) entry. Ca^+2^ ions bind Calmodulin (CaM) and activate membrane bound AC which is also activated by β2 adrenergic receptors (βAR) and prostacyclin receptors (IPR). AC catalyzes the conversion of adenosine monophosphate (AMP) to cyclic adenosine monophosphate (Chu et al.) that activates Protein kinase A (PKA). Phosphodiesterases (PDE) facilitate the conversion of cAMP to AMP or cyclic guanosine monophosphate (cGMP) to guanosine monophosphate (GMP).

In SCD, RBC ATP level is reduced, and ATP depletion is associated with increased number of irreversibly sickled RBCs (Jensen et al., 1973; Banerjee and Kuypers, 2004). RBCs are known to release ATP in a response to mechanical stress. Inactivation of AC/PKA signaling pathway attenuates ATP release and improves mechanical stress responses of RBC by increasing deformability (Sprague et al., 2001). Therefore, one may suggest that PKA inhibition could be beneficial for the rheological properties of sickle RBCs. Since PKA activity is dependent on cAMP levels, AC activity is also important for PKA-dependent processes by the conversion of AMP to cAMP. Sickle RBCs are known to contain more than 4-fold cAMP levels compared to healthy RBCs (Hines et al., 2003). The stimulation of AC by Forskolin has been shown to further increase cAMP levels in sickle RBCs, which resulted in increased adhesiveness to laminin (Hines et al., 2003). Our results in the present study demonstrate that AC stimulation by Forskolin lead to a reduction of RBC deformability, plausibly by the enhancement of cAMP levels. Interestingly, few studies showed that Forskolin increased deformability of healthy RBCs (Muravyov et al., 2009; Muravyov and Tikhomirova, 2013). However, Semenov et al. (2019) demonstrated that the improvement of RBC deformability by Forskolin would be dependent on both the dosage of the drug and the level of shear stress. Similarly, we showed that Forskolin increased RBC deformability before the implementation of constant shear stress; however, RBC deformability was decreased after the 5 Pa application. Physiologically relevant shear stress increases deformability in healthy RBCs (Meram et al., 2013). The impairment of RBC deformability by Forskolin after 5 Pa application in SCD patients could be explained by the calcium influx through shear stress sensing mechanisms that could stimulate AC and lead to the elevation of intracellular cAMP levels (Figure 8). Several nonselective cationic ion channels are present at the membrane of RBCs and can be activated by shear stress, resulting in increased Ca^2+^ influx (Kaestner et al., 2020; Egée and Kaestner, 2021; Nader et al., 2023). The decrease of RBC deformability through AC stimulation was well observed at high shear stresses suggesting that the effect of Forskolin is shear stress dependent.

The inhibition of AC and PKA by SQ22536 and H89, respectively, significantly increased RBC deformability of SCD patients. The improving effects of H89 and SQ22536 on RBC deformability were more pronounced than the effects of Forskolin, which can be observed in SS_1/2_, EI_max_, and SS_1/2_:EI_max_ parameters. The SS_1/2_ parameter provides a global index of RBC deformability, while EI_max_ indicates the limiting elongation index at infinite shear stress (Baskurt et al., 2009). EI_max_ may be affected by cell shape and membrane properties, however, this parameter is not impacted by cytoplasmic viscosity (Baskurt et al., 2009). A reduced SS_1/2_ often indicates improved RBC deformability, however, this might also be related to a reduced EI_max_. Therefore, SS_1/2_:EI_max_ ratio should be considered and reflects the dependence of EI on SS independent of EI_max_ alterations (Baskurt and Meiselman, 2013). In our previous study, we also showed that the inhibition of AC/PKA signaling pathway resulted in a rise of RBC deformability in SCD patients (Ugurel et al., 2019). AC inhibition reduces cAMP levels and suppresses PKA activation. PKA targets several membrane proteins and regulates the activities of ion channels. PKA phosphorylates dematin, Protein 4.1 and adducin in RBC membrane that promotes the dissociation of the spectrin network and reduces membrane stability (Cohen and Gascard, 1992; Koshino et al., 2012; Chen et al., 2013). PKA also targets CFTR channel in RBC and regulates Cl efflux (Decherf et al., 2007). This mechanism could affect cell volume with a significant impact on RBC deformability. On the other hand, the enhancement of AC/PKA signaling increases the adhesion of sickle RBCs to endothelium through Lu/BCAM adhesion molecule (Zennadi et al., 2004; Gauthier et al., 2005). Abnormal adherence of sickle RBCs to endothelial cells was postulated to be important in the initiation and progression of vaso-occlusive crises (Hebbel, 1997; Kaul and Fabry, 2004). AC/PKA signaling pathway also activates ERK1/2 signaling molecule, which phosphorylates ICAM-4 adhesion receptor on RBCs and promotes sickle cell adhesion (Zennadi et al., 2012). This mechanism of action could be reversed by blocking AC or PKA activities that suppresses sickle RBC adhesion to the endothelium (Zennadi et al., 2012).

AC induced synthesis of cAMP requires the stimulation of G protein coupled receptors (GPCR) which includes β2-adrenergic receptors (βAR) and prostacyclin receptors (IPR) in RBCs (Sprague et al., 2008). Intracellular signal transduction is mediated by increased levels of cAMP, which is then carried out by PKA (Figure 8). However, the signal can be attenuated due to the hydrolysis of cAMP by phosphodiesterases (PDEs) (Baillie, 2009). Although 11 different PDE families are present in various cell types, only 6 of them (PDE1, 2, 3, 4, 5, and 9) are defined in mature RBCs or erythroid precursors (Almeida et al., 2008; Adderley et al., 2011). Non-selective inhibition of PDE by Pentoxifylline in the present study significantly increased the deformability of RBCs from SCD patients, both before and after the application of a constant shear stress for prolonged time, which confirms previous findings (Ugurel et al., 2019) but contrast with another study (Cummings and Ballas, 1990). A previous case report in the late seventies showed that Pentoxifylline treatment in a patient with SCD and frequent vaso-occlusive crises improved RBC deformability and decreased blood viscosity (Keller and Leonhardt, 1979). A recent study demonstrated that the elastic modulus of RBC was decreased by Pentoxifylline treatment *in vivo*, which improved blood flow in subjects with cerebrovascular and peripheral arterial diseases (Aifantis et al., 2019). Pentoxifylline is postulated to increase intracellular ATP concentrations, decrease Ca^2+^ concentrations by activation of the Ca^2+^—Mg^2+^ ATPase and calmodulin, and increase the phosphorylation of proteins into the RBC membrane (Schubotz and Mühlfellner, 1977; Aifantis et al., 2019), which could increase RBC deformability. We previously demonstrated that tyrosine phosphorylation of membrane proteins was increased by the application of Pentoxifylline *in vitro* and was accompanied by an increase of RBC deformability in healthy donors (Ugurel et al., 2022). However, several clinical trials conducted in SCD did not demonstrate any clinical improvement induced by Pentoxifylline, as a preventive molecule, in patients with frequent vaso-occlusive crises (Sherer and Glover, 2000). In contrast, a study demonstrated that the use of Pentoxifylline during the acute phase of vaso-occlusive crisis could be helpful for faster recovery (Poflee et al., 1991). Nevertheless, its clinical impact seems to be rather limited (Sherer and Glover, 2000).

According to the present study, the inhibition of PDE1 by Vinpocetine significantly increased RBC deformability of SCD patients. Vinpocetine was previously shown to improve RBC deformability in healthy subjects and stroke patients (Hayakawa, 1992; Muravyov and Tikhomirova, 2015). This selective inhibitor of PDE1 has no effect on the increase of cAMP in either βAR or IPR pathway suggesting that PDE1 is involved in hydrolysis of cGMP in RBCs (Adderley et al., 2009). The improvement of RBC deformability from SCD patients by PDE1 inhibition could be explained by the elevation of intracellular cGMP levels. PDE4 inhibition by Rolipram decreased RBC deformability from SCD patients. The alterations seem to be more pronounced after the 5 Pa application showing that mechanical stress responses of sickle RBCs are deteriorated by PDE4 inhibition. PDE4 is known to be responsible for hydrolyzing cAMP in healthy RBCs. The selective inhibition of PDE4 by Rolipram increased cAMP levels in RBCs with the stimulation of adrenergic pathway by isoproterenol (Adderley et al., 2009). Rolipram was shown to increase the deformability in healthy RBCs (Muravyov and Tikhomirova, 2015), however we have demonstrated that this drug decreased RBC deformability from SCD patients, particularly at high shear stress. We suspect that the responses of sickle RBCs to mechanical stress would be altered due to the impairment in cAMP signaling. Furthermore, prolonged hypoxia in transgenic SCD mice increased PDE4 levels in lung tissue, which was reversed by Rolipram, preventing the development of pulmonary arterial hypertension (De Franceschi et al., 2008). Rolipram was also shown to reduce ischemic/reperfusion liver injury in transgenic SCD mice most likely by inducing over-expression of Nos3 and reducing vascular activation (Filippini et al., 2008). Although these studies revealed the protective effects of PDE4 inhibition for ischemic injury and hypertension in a SCD model, they did not investigate the efficacy of PDE4 on sickle RBCs. Another PDE family investigated in the present study was PDE3, which hydrolyzes both cAMP and cGMP in a complex manner. The hydrolysis of cGMP by PDE3 can inhibit the hydrolysis of cAMP in various cell types (Degerman et al., 1997; Bender and Beavo, 2006). PDE3 inhibition in RBC had no effect on cAMP levels stimulated by βAR, however PDE3 selectively regulates cAMP synthesis when stimulated by IPR signaling (Hanson et al., 2008; Adderley et al., 2009). We did not initially stimulate βAR or IPR pathways; however, we studied the effects of PDE3 inhibition on sickle RBCs in native conditions. Accordingly, the inhibition of PDE3 by Milrinone did not significantly alter RBC deformability in SCD patients suggesting sickle RBC deformability is not modulated in an IPR dependent way.

## 5 Conclusion

Although our study showed an effect of most of the drugs used on RBC deformability from SCD patients, the potential clinical relevance is unknown. Most of the changes observed in RBC deformability are rather small in comparison with the effects of other drugs currently used in the context of SCD, such as Hydroxyurea (Charache et al., 1995; Lemonne et al., 2015) or Voxelotor (Dufu et al., 2018; Migotsky et al., 2022), and where clinical benefits have been reported. The only change we noted, which seems to be physiologically relevant, is the decrease of RBC deformability observed when stimulating AC with Forskolin and blocking PDE4 by Rolipram. Following the application of prolonged shear stress, both drugs reduced the RBC deformability of SCD patients, indicating that the effects of these two compounds might be shear-dependent. However, the results from the present study are preliminary and limited to a small sample size: further studies are needed with a larger group of patients to identify the factors that could be involved in the variability of the responses. Developing drugs targeting the AC signaling pathway mediated by βAR receptors could have potential clinical application but further studies are needed. Nevertheless, the Figure 7 shows the example of a patient with SCD whose *in-vitro* responses to most of the drugs were highly significant from a physiological/rheological point of view, suggesting that the effects of the different drugs tested in the present study are highly variable from one patient to another and that some patients could benefit from one drug while other not. Differential responses of sickle RBCs might be due to variable expression levels of PDEs in each patient, as well. Our next studies will include the quantification of PDEs in RBC samples from SCD patients. Further large *in-vitro* studies are needed to identify why some sickle RBCs could respond more than others.

## Data Availability

The raw data supporting the conclusion of this article will be made available by the authors, without undue reservation.
